# Convallaria Majalis: A Proving

**Published:** 1884-05

**Authors:** Irvin J. Lane

**Affiliations:** Sing Sing, N. Y.


### CONVALLARIA MAJALIS: A PROVING.

IRVIN J. LANE, M. D., SING SING, N. Y.

The provers were I. J. Lane, Mrs. C. E. Lane, and James A. Vansant. The fluid extract was used in five to twenty drop doses. The symptoms in italic are those we both had when taking Convallaria majalis, excepting those of the female organs; those were very marked, or occurred a number of times.

MIND.—Depression; mind wanders from subject when reading; feels irritable when asked a question.

HEAD.—Vertigo in the morning, with faint feeling; dull headache, commencing in vertex and extending to temporal region; *dull feeling in the head;* dull heavy ache over the eyes; dull aching in right eye and temple, with pain from eye extending over top of head and down right side of neck about every fifteen minutes.

EYES.—On going from a dark room into the light sees gray-colored spots about three inches square in different parts of the room; all letters look alike when reading; sees small words before the beginning of a sentence; the letter P is substituted for other letters; upper eyelids feel heavy when looking up; dull aching pain in right eye.

EAR.—Pulsating pain in left ear, with heat; pulsating pain in right middle ear; when swallowing tympanum of right ear seems to bulge out, relieved by pressing on temporal artery in front of ear.

FACE.—Smoked color, after exercise; looked pinched.

THROAT.—*Dry suffocating sensation,* as if she could not get her breath.

STOMACH.—*Eructations tasting of fat; sometime after eating eructations tasting of food;* nausea and faint feeling in the morning, followed by vomiting a small quantity of a clear substance tasting like phlegm; nausea after eating and exercise, with vomiting of mucus, tasting like slime from oysters; increased appetite. Symptoms relieved by eating, but returning again soon.

ABDOMEN.—Dull colic-like pain in left iliac region, relieved by micturition; colic-like pains in umbilical region, come suddenly and pass off slowly; *uneasy sensation, causing desire for stool; colic in lower part of abdomen; sore aching pain in hypogastric region; sore aching pain in lower part of abdomen, causing me to hold my breath; feel sleepy during pains; coughing causes sore pain in hypogastric region;* colic-like pains commencing on right side, and going to left; sensation as if she was filling up, causing dyspnœa; abdomen seems larger than it really is, although somewhat distended when sitting; *dull aching paroxysmal pain, causing desire for stool, relieved by stool; laughing or coughing causes a sore feeling in lower part of abdomen.*

STOOL AND ANUS.—Sore burning pain at anus during stool; *uneasy sensation in abdomen, causing desire for stool; dull aching pain in hypogastric region, with feeling of fullness and desire for stool, relieved by stool;* slight diarrhœa; diarrhœa between two and six P. M., offensive odor, like decayed meat, light brown color; slight tenesmus; dull grumbling pain in bowels as if they would move; sensation of an abundance of flatulence in abdomen that would pass at any time but can retain it; rectum full of gas, not relieved by passing flatus; uneasy feeling in hypogastric region, with colic; would last about fifteen minutes, then there would be an urgent call for stool, which would relieve.

URINARY ORGANS.—*Some sugar and phosphates;* frequent desire to urinate, passing only a small quantity at a time; urine seemed scalding hot, but left no smarting.

THORAX.—*Sharp pains in right or left nipple;* sharp radiating pains beneath the upper part of sternum.

FEMALE ORGANS.—*Labor-like pains in sacro-iliac synchondrosis, as in first stage of labor; bearing down sensation worse on right side; sleepy between the pains; pains come quick and pass off slowly;* labor-like pains extending along inner side of right thigh; all the above pains aggravated by motion, sitting up straight or leaning back; relieved by bending forward. Pain commencing in anterior part of abdomen on right side, as in second stage of labor, but extending up higher; sensation as if a large cord extended from sacro-iliac synchondrosis to inguinal region, which was pulled down by the pelvic organs; worse on right side. Uterus seems descended and retroverted, the fundus of uterus pressing on the rectum, causing a very hard, aching pain in rectum and anus. The pain was described as continuous and unbearable; labor-like pains extending up sides and back, causing nausea and faintness, with hunger; pain relieved when lying on back, but then there was a sensation as if a fœtus at eight months was kicking in abdomen; aggravated by being on her feet; *sensation in right breast as if milk were coming in breast, followed by sharp, stinging pains centering in the nipple; intense itching, commencing at orifice of vagina and gradually extending to meatus urethræ, and all over the labia, with great hyperæmia but no eruption; it was almost unbearable, causing a feeling as if she must weep; aggravated by the slightest motion of legs; relieved by applying cold water. Raising head from pillow in the morning, had a feeling like morning sickness; relieved by vomiting a small quantity of a clear substance, tasting like phlegm.* Eructations during the labor-like pains.

RESPIRATORY ORGANS.—Dyspnœa, caused by a sensation as if she were filling up in the abdomen; desire to take a deep breath when sitting.

HEART AND PULSE.—*Heart's action very weak; pulse very weak and compressible;* dicrotic pulse; heart-sounds feeble; anæmic murmur heard over jugular vein; *pulse imperceptible, with hand extended above the head;* sensation as if the heart had been palpitating; when exercising, heart would flutter for about a minute, then the face would get red, and then there was a sensation as if the heart stopped beating, starting again very suddenly, causing a faint feeling; *pulse full, compressible, and inter-*

*mittent.* These symptoms under heart and pulse are very characteristic of the drug.

NECK AND BACK.—*Dull aching, or sore pain in lumbar region; aggravated by sitting up straight or leaning back; sharp or dull pain under lower angle of scapula, ameliorated by bending the shoulders back;* sharp pains in region of right kidney; at two P. M., dull aching pain between lower angles of scapula, extending up and down the spine; pressive aching pain under lower angle of right scapula; constant gaping and yawning after the pain.

LOWER LIMBS.—Cramps in inner side of right thigh, relieved by pressing foot against something; aching pain in ankles; knees sore while walking; cramps in calf of right leg while sitting, relieved by standing; passed off quickly, returned after sitting down; when first bearing weight on her foot after lying down they were numb, then a sensation as if the soles of feet were full of needles (or asleep).

UPPER LIMBS.—Rheumatic pains in left elbow joint, at night, on exposure to cold; ameliorated by motion; hand numb, and went to sleep on holding it above the head; aching pains in wrists.

GENERALITIES.—Pains leave a sore feeling; *feel sleepy between the pains;* faint feeling from slight cause; hand went to sleep when extended above the head; sharp pains in nipples; sensation as if getting fat inside, filling her up; sleepy and all tired out, as from bodily exertion; sick feeling all over; great prostration; feeling as if just recovering from a long sickness.

SKIN.—Blotches like mosquito bites, commencing on anterior part of thigh; great itching, not relieved by scratching; worse on exposure to the air; itching on anterior part of thigh, but no eruption.

SLEEP.—Sleepy on going in a warm room from open air, *sleepy while in warm room; awoke about* two A. M., *be very restless for some time, then go to sleep again,* and sleep until about five A.M., then awake again, and would be very restless until morning, restless after sleeping between the pains.

FEVER.—Burning, hot feeling, over the body after midnight; temperature decreased; perspiration over whole body; short paroxysms of hot, suffocating sensation, with perspiration over body.

AGGRAVATIONS.—*Sitting up straight or leaning back,* laughing or coughing every day.

AMELIORATION.—*In open air,* by exercise, standing, lying down, *leaning forward while sitting,* eating.

---

NOTE.—Dr. Lane writes us that he hopes to make further provings, using potencies. These are necessary to bring out the characteristics of a drug.—ED.

